# Supplementary material for: Association Between Previous Statin Use and Alzheimer’s Disease: A Nested Case-Control Study Using a National Health Screening Cohort
Source: Brain Sci. 2021 Mar 20;11(3):396. doi: 10.3390/brainsci11030396 (PMC8003839; doi:10.3390/brainsci11030396)
Supplement: Supplementary file 1 [file brainsci-11-00396-s001.pdf]

## Supplementary Material

**Supplementary File S1.** Description of diagnosis of dementia

Dementia patients were considered if they were diagnosed with Alzheimer's disease (ICD-10 code: G30) or dementia in Alzheimer's disease (F00). We selected only the participants who were treated ≥2 times. 123,025 participants were ≥65 years old in 2012 in our previous studies using this national sample cohort. Among them, 9,740 (7.9%) participants were categorized as dementia according to our methods (5.4% [n =2,758] in male, 9.7% [n= 6,982] in female).

We could compare these results of central dementia center of Korea ([www.nid.or.kr](http://www.nid.or.kr)) which is controlled by Ministry of Health and Welfare of Korea. The earliest data was in 2012, and it was available in ≥65 years old. According to their data, the prevalence of dementia (Alzheimer's disease, and others) except vascular dementia were 7.63% (4.47% in male, 9.85% in female).

**Supplementary Tables S1.** Subgroup analyses of odds ratios (95% confidence interval) for the day of statin use per year in Alzheimer's disease patients compared to control participants according to obesity, smoking, alcohol consumption, total cholesterol, blood pressure, and fasting blood glucose

| Characteristics | Odds ratios | | | | | | *p* value for interaction |
|---|---|---|---|---|---|---|---|
| | Model 1[b] | *p* value | Model 2[c] | *p* value | Model 3[d] | *p* value | |
| Obesity | | | | | | | 0.587 |
| Underweight (n = 3,877) | 1.23 (1.04-1.44) | 0.014[a] | 1.08 (0.90-1.31) | 0.410 | 1.08 (0.89-1.31) | 0.433 | |
| Normal weight (n = 31,877) | 1.03 (0.98-1.08) | 0.255 | 0.96 (0.91-1.01) | 0.104 | 0.95 (0.90-1.00) | 0.044[a] | |
| Overweight (n = 21,640) | 1.01 (0.96-1.06) | 0.800 | 0.97 (0.91-1.03) | 0.317 | 0.94 (0.89-1.00) | 0.067 | |
| Obese (n = 28,466) | 1.01 (0.97-1.06) | 0.576 | 0.97 (0.93-1.02) | 0.276 | 0.96 (0.91-1.01) | 0.080 | |
| Smoking | | | | | | | 0.773 |
| Nonsmoker (n = 68,124) | 1.00 (0.98-1.03) | 0.768 | 0.96 (0.92-0.99) | 0.009[a] | 0.96 (0.92-0.99) | 0.011[a] | |
| Past smoker and current smoker (n = 17,736) | 0.99 (0.93-1.05) | 0.648 | 0.95 (0.89-1.02) | 0.155 | 0.94 (0.88-1.01) | 0.079 | |
| Alcohol consumption | | | | | | | 0.257 |
| <1 time a week (n = 64,424) | 1.02 (0.99-1.05) | 0.269 | 0.96 (0.93-0.99) | 0.020[a] | 0.96 (0.92-0.99) | 0.013[a] | |
| ≥1 time a week (n = 21,436) | 0.96 (0.90-1.01) | 0.107 | 0.93 (0.87-0.99) | 0.031[a] | 0.94 (0.88-1.00) | 0.063 | |
| Total cholesterol (mg/dL) | | | | | | | 0.746 |
| <200 (n = 47,207) | 0.99 (0.96-1.02) | 0.589 | 0.93 (0.89-0.97) | < 0.001[a] | 0.94 (0.90-0.98) | 0.003[a] | |
| ≥200 to < 240 (n = 26,825) | 1.03 (0.98-1.09) | 0.279 | 0.96 (0.9-1.02) | 0.160 | 0.94 (0.88-1.00) | 0.053 | |
| ≥240 (n = 11,828) | 0.99 (0.92-1.07) | 0.839 | 0.99 (0.91-1.08) | 0.884 | 0.97 (0.89-1.06) | 0.483 | |
| Blood pressure (mmHg) | | | | | | | 0.079 |
| SBP <140 and DBP <90 (n = 57,161) | 1.01 (0.98-1.04) | 0.583 | 0.95 (0.92-0.99) | 0.012[a] | 0.96 (0.92-0.99) | 0.019[a] | |

| | | | | | | | |
|---|---|---|---|---|---|---|---|
| SBP ≥140 or DBP ≥90 (n = 28,699) | 0.98 (0.94-1.03) | 0.446 | 0.96 (0.91-1.01) | 0.118 | 0.95 (0.90-1.00) | 0.051 | |
| Fasting blood glucose (mg/dL) | | | | | | | 0.979 |
| <100 (n = 48,871) | 0.98 (0.95-1.02) | 0.414 | 0.94 (0.90-0.98) | 0.007[a] | 0.94 (0.90-0.98) | 0.008[a] | |
| ≥100 (n = 36,989) | 0.99 (0.96-1.03) | 0.678 | 0.97 (0.93-1.01) | 0.127 | 0.96 (0.92-1.01) | 0.081 | |

Note: CCI–Charlson comorbidity index; DBP–diastolic blood pressure; SBP–systolic blood pressure.[a] Conditional logistic regression, Significance at $p < 0.05$; [b] Models were stratified by age, sex, income, and region of residence; [c] A model 1 was adjusted for dyslipidemia history, total cholesterol, SBP, DBP, and fasting blood glucose; [d] A model 2 was adjusted for model 1 plus obesity, smoking, alcohol consumption, and CCI scores.
